# VEGF_189_ binds NRP1 and is sufficient for VEGF/NRP1-dependent neuronal patterning in the developing brain

**DOI:** 10.1242/dev.115998

**Published:** 2015-01-15

**Authors:** Miguel Tillo, Lynda Erskine, Anna Cariboni, Alessandro Fantin, Andy Joyce, Laura Denti, Christiana Ruhrberg

**Affiliations:** 1 UCL Institute of Ophthalmology, University College London, 11-43 Bath Street, London EC1V 9EL, UK; 2 School of Medical Sciences, Institute of Medical Sciences, University of Aberdeen, Aberdeen AB25 2ZD, UK; 3 University of Milan, Department of Pharmacological and Biomolecular Sciences, Via Balzaretti 9, Milan 20133, Italy

**Keywords:** Vascular endothelial growth factor (VEGF), VEGF189, Neuron, Neuropilin, Mouse

## Abstract

The vascular endothelial growth factor (VEGFA, VEGF) regulates neurovascular patterning. Alternative splicing of the *Vegfa* gene gives rise to three major isoforms termed VEGF_121_, VEGF_165_ and VEGF_189_. VEGF_165_ binds the transmembrane protein neuropilin 1 (NRP1) and promotes the migration, survival and axon guidance of subsets of neurons, whereas VEGF_121_ cannot activate NRP1-dependent neuronal responses. By contrast, the role of VEGF_189_ in NRP1-mediated signalling pathways has not yet been examined. Here, we have combined expression studies and *in situ* ligand-binding assays with the analysis of genetically altered mice and *in vitro* models to demonstrate that VEGF_189_ can bind NRP1 and promote NRP1-dependent neuronal responses.

## Abstract (precis)

**Summary**: Although VEGF165 was thought to be the sole VEGF isoform acting through neuropilin 1 (NRP1), VEGF189 also binds to and signals through NRP1 in several types of developing mouse neurons.

## INTRODUCTION

**Fig. 1.**
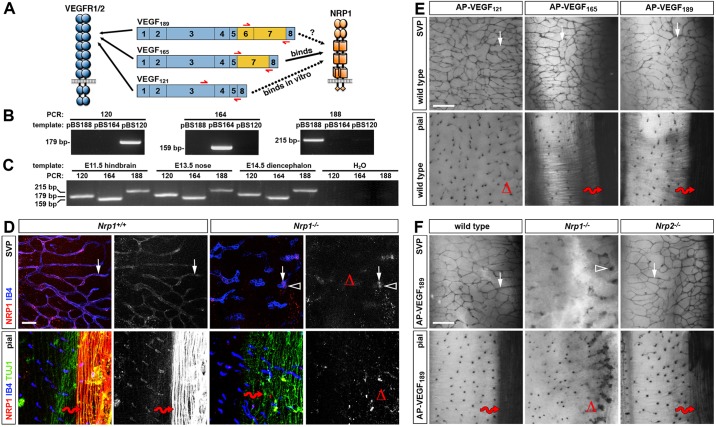
**VEGF_189_ is expressed in developing mouse tissues and binds NRP1 in the developing hindbrain.** (A) Current knowledge of VEGF isoform binding to their receptors. All isoforms bind VEGFR1/2, whereas only VEGF_165_ is known to bind NRP1. VEGF_121_ can bind NRP1 with low affinity *in vitro*, but whether this association occurs *in vivo* has not been shown. Moreover, it has not been shown whether VEGF_189_ binds NRP1 *in vivo*. Red arrows below each isoform indicate the position of oligonucleotide primers used for RT-PCR in B. (B) *Vegfa* isoform-specific oligonucleotide primers for RT-PCR were validated with pBlueScript vectors (pBS) containing mouse *Vegfa120*, *Vegfa164* or *Vegfa188* cDNA, respectively. (C) RT-PCR analysis of the indicated tissues shows that *Vegfa120* (179 bp), *Vegfa164* (159 bp) and *Vegfa188* (215 bp) are co-expressed. (D) Whole-mount staining of E12.5 wild-type hindbrains for NRP1 and TUJ1 together with IB4; single NRP1 channels are shown in grey scale adjacent to each panel. The white arrows indicate IB4-positive vessels; the arrowhead indicates nonspecific NRP1 staining of blood cells inside mutant vessels; the red wavy arrows indicate TUJ1-positive axons; open triangles indicate absent NRP1 staining in subventricular plexus (SVP) vessels and pial axons. Scale bar: 200 μm. (E,F) AP-VEGF_121_, AP-VEGF_165_ and AP-VEGF_189_ binding to E12.5 wild-type hindbrains (E) and AP-VEGF_189_ binding to E12.5 *Nrp1^−/−^* and *Nrp2^−/−^* hindbrains (F). The white arrows indicate VEGF binding to vessels; the red wavy arrows indicate binding to axons; the open triangles indicate absence of VEGF_121_ binding to wild-type axons in E and absence of VEGF_189_ binding to axons in *Nrp1^−/−^* hindbrains in F. The arrowhead indicates vascular tufts. Scale bars: 25 μm.

Vascular endothelial growth factor A (VEGFA, VEGF) is a potent inducer of blood vessel growth, but also has essential roles in neurodevelopment (Mackenzie and Ruhrberg, 2012). In humans, VEGF is encoded by a single gene (*VEGFA*) of eight exons that is alternatively spliced into isoforms, the major ones containing 121, 165 and 189 amino acid residues and therefore termed VEGF_121_, VEGF_165_ and VEGF_189_, respectively (Fig. 1A; Koch et al., 2011). The alternatively spliced exons 6 and 7 encode domains that enable extracellular matrix (ECM) binding and additionally mediate differential binding to VEGF receptors. All VEGF isoforms bind the receptor tyrosine kinases VEGFR1 (FLT1) and VEGFR2 (KDR, FLK1), whereas the non-catalytic receptors neuropilin (NRP) 1 and NRP2 are VEGF isoform-specific receptors that preferentially bind VEGF_165_ over VEGF_121_ (Fig. 1A; Gluzman-Poltorak et al., 2000; Soker et al., 1998). Unexpectedly, recent studies showed that VEGF binding to NRP1 is largely dispensable for embryonic angiogenesis (Fantin et al., 2014). By contrast, VEGF signalling through NRP1 has multiple roles in neurodevelopment, including guiding migrating facial branchiomotor (FBM) neurons in the hindbrain (Schwarz et al., 2004), promoting the survival of migrating gonadotropin-releasing hormone (GnRH) neurons (Cariboni et al., 2011) and enhancing the contralateral projection of retinal ganglion cell (RGC) axons across the optic chiasm (Erskine et al., 2011).

To demonstrate roles for VEGF binding to NRP1 in neurons, prior studies used *Vegfa^120/120^* mice, which express VEGF_120_, the murine equivalent of VEGF_121_, but lack VEGF_164_ and VEGF_188_, corresponding to human VEGF_165_ and VEGF_189_, respectively (Carmeliet et al., 1999). *Vegfa^120/120^* mice phenocopy the defects of NRP1 knockouts in FBM neuron migration, GnRH neuron survival and RGC axon guidance (Cariboni et al., 2011; Erskine et al., 2011; Schwarz et al., 2004). In all three systems, VEGF signalling was attributed to the activity of VEGF_165_ because it evokes appropriate neuronal responses in tissue culture models (Cariboni et al., 2011; Erskine et al., 2011; Schwarz et al., 2004), and because the ability of NRP1 to bind VEGF_165_ is well established (Fantin et al., 2014; Soker et al., 1998). However, *Vegfa^120/120^* mutants lack VEGF_188_ in addition to VEGF_164_. Yet, it has never previously been examined whether VEGF_189_ can also function as a NRP1 ligand *in vivo*. Moreover, it is not known whether VEGF_121_ can bind NRP1 in a physiologically relevant context, even though it has been suggested that the exon 8 domain, which is present in all major VEGF isoforms, including VEGF_121_, can mediate NRP1 binding *in vitro* (Jia et al., 2006; Pan et al., 2007; Parker et al., 2012).

Here, we have generated alkaline phosphatase (AP)-conjugated VEGF isoforms for *in situ* ligand-binding assays (Fantin et al., 2014) to examine whether VEGF_121_ or VEGF_189_ can bind NRP1 *in vivo*, as previously reported for VEGF_165_. Our studies demonstrate that VEGF_189_ binds NRP1-expressing axon tracts in intact hindbrain tissue, but that VEGF_121_ is unable to do so. We further show that VEGF_188_ is co-expressed with the other isoforms during VEGF/NRP1-dependent FBM migration, GnRH neuron survival and RGC axon guidance, and that VEGF_188_ is sufficient to control all three processes, whereas VEGF_120_ is not. We conclude that VEGF_188_ effectively binds NRP1 and has the capacity to evoke NRP1-dependent signalling events, similar to VEGF_164_. Considering that VEGF_189_ has the highest affinity for ECM and therefore tissue retention amongst the VEGF isoforms, future research may therefore wish to consider the mechanistic contribution and therapeutic potential of this understudied VEGF isoform.

## RESULTS AND DISCUSSION

### VEGF_188_ is co-expressed with VEGF_120_ and VEGF_164_ in developing hindbrain, nose and diencephalon, and binds axons in a NRP1-dependent fashion

Because prior studies implicated VEGF signalling through NRP1 in FBM neuron migration in the hindbrain, GnRH neuron survival in the nose and RGC axon guidance in the diencephalon (Cariboni et al., 2011; Erskine et al., 2011; Schwarz et al., 2004), we asked which *Vegfa* isoforms were expressed in these developmental contexts. For this experiment, we designed isoform-specific primers that can distinguish the *Vegfa120*, *Vegfa164* and *Vegfa188* mRNA species by reverse transcription (RT)-PCR (Fig. 1A,B; supplementary material Fig. S1A). This analysis demonstrated that all three isoforms were co-expressed during relevant periods of VEGF/NRP1-dependent neurodevelopment in mice (Fig. 1C).

Because prior studies of VEGF binding to NRP1 have not examined whether VEGF_189_ or VEGF_121_ can bind NRP1 *in vivo*, we used the mouse hindbrain as a physiologically relevant model to compare the ability of the three major VEGF isoforms to bind NRP1 in a tissue context. We first performed immunostaining with a validated antibody for NRP1 (Fantin et al., 2010) to confirm that NRP1 localises to blood vessels in wild-type, but not NRP1 knockout, hindbrains (Fig. 1D; note unspecific staining of blood in the dilated vessels of mutants). Immunolabelling also confirmed NRP1 expression in TUJ1-positive dorsolateral axons on the pial side of wild-type, but not mutant, hindbrains (Fig. 1D; supplementary material Fig. S1B). *Nrp1*^−/−^ hindbrains showed some defasciculation of these dorsolateral axons, but they were still clearly present in the mutant, suggesting that this are a suitable model to examine VEGFA isoform binding to NRP1.

To compare the binding properties of VEGF_121_, VEGF_165_ and VEGF_189_, we fused each isoform to AP and performed *in situ* ligand binding assays on E12.5 hindbrains. As expected, all three isoforms bound vessels (Fig. 1E), because they express the pan-VEGF isoform receptor VEGFR2 (Lanahan et al., 2013). We next examined binding to dorsolateral axons, because they express NRP1, but lack VEGFR2 (Lanahan et al., 2013). Both VEGF_165_ and VEGF_189_ bound these axons, whereas VEGF_121_ did not (Fig. 1E). These observations indicate that all VEGF isoforms are capable of binding VEGFR2/NRP1-positive vessels. By contrast, only VEGF_165_ and VEGF_189_, but not VEGF_121_, bound NRP1-expressing axons lacking VEGFR2, consistent with the previously reported 10-fold lower affinity of VEGF_121_ for NRP1 *in vitro* (Parker et al., 2012). The finding that VEGF_121_ does not bind endogenous neuronal NRP1 at detectable levels also agrees with prior genetic studies, which showed that VEGF_120_ is unable to compensate for VEGF_164_ in FBM, RGC and GnRH neurons (Cariboni et al., 2011; Erskine et al., 2011; Schwarz et al., 2004). Thus, low-affinity binding of VEGF_121_ to NRP1, even though previously observed *in vitro*, is unlikely to be relevant *in vivo*, at least in a neuronal context.

We next confirmed that axonal VEGF_189_ binding is NRP1 dependent. The AP ligand-binding assay showed that VEGF_189_ bound vessels (Fig. 1F) in *Nrp1*-null mutant hindbrains with their characteristic vascular tufts (Fantin et al., 2013a). Strikingly, AP-VEGF_189_ failed to bind axons in *Nrp1*-null hindbrains, similar to AP-VEGF_165_ (Fig. 1F). VEGF_189_ can therefore bind axons in a NRP1-dependent fashion. By contrast, loss of NRP2 (Giger et al., 2000) did not abolish VEGF_189_ binding (Fig. 1F). Taken together, the ligand binding assays of intact hindbrain tissue show that NRP1 serves as a neuronal receptor for VEGF_165_ and VEGF_189_, but not for VEGF_121_.

### VEGF_188_ is sufficient for the NRP1-dependent migration of FBM neurons

**Fig. 2.**
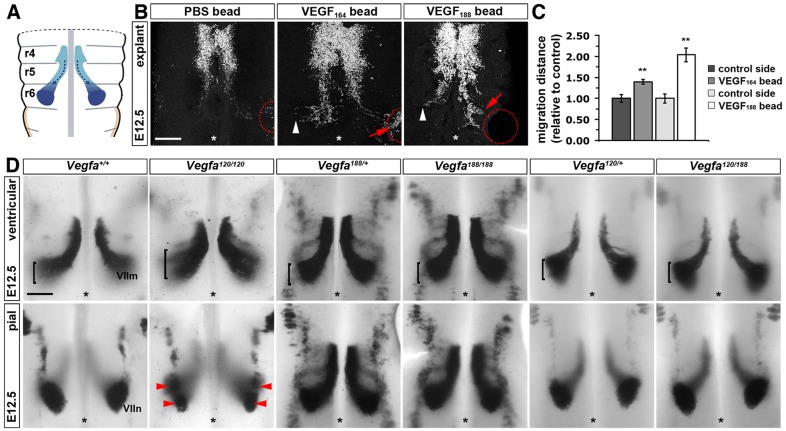
**VEGF_188_ is sufficient for FBM neuron migration.** (A) Schematic representation of FBM neuron migration in the mouse. (B) ISL1 staining of E12.5 hindbrain explants containing implanted heparin beads soaked in PBS (*n*=10) or PBS containing VEGF_164_ (*n*=10) or VEGF_188_ (*n*=6). Red dotted circles indicate the position of heparin beads; white arrowheads indicate normal migration; red arrows indicate migration towards heparin beads; asterisks indicate the midline. Scale bar: 200 µm. (C) Distance migrated by FBM neurons. Migration distance was quantified as migration away from r5 territory on the hindbrain side with a bead relative to the control half of the same hindbrain; mean±s.e.m. control 1±0.09 versus VEGF_164_ bead 1.39±0.05; control 1±0.11 versus VEGF_188_ bead 2.04±0.17; **P<0.01, VEGF compared with control (*t*-test). (D) Whole-mount *Isl1 in situ* hybridisation of E12.5 hindbrains of the indicated genotypes detects migrating FBM neurons (VIIm) (control, *n*=10; *Vegfa^120/120^*, *n*=6; *Vegfa^188/188^, n*=4; *Vegfa^120/188^*, *n*=5). Brackets indicate the width of the neuronal stream on the ventricular side; red arrowheads indicate dumbbell-shaped nuclei on the pial side; asterisks indicate the midline*.* Scale bar: 25 µm.

*Vegfa* is a haploinsufficient gene for which deletion of just one allele results in early embryonic lethality due to a complete failure of blood vessel formation (Carmeliet et al., 1996; Ferrara et al., 1996). However, retention of any one of the major VEGF isoforms rescues this severe phenotype and instead gives rise to more subtle neuronal and vascular phenotypes (Ruhrberg et al., 2002; Stalmans et al., 2002). Understanding the receptor-binding properties of the VEGF isoforms has therefore become a priority in the field. We first examined if VEGF_188_ can substitute for VEGF_164_ in FBM neuron guidance with an established hindbrain explant assay in which implanted beads provide exogenous VEGF, and FBM neuron migration is visualised by immunolabelling with the motor neuron marker ISL1 (Schwarz et al., 2004; Tillo et al., 2014). Agreeing with previous observations, FBM neurons were attracted to VEGF_164_, but not to control beads lacking growth factors (Fig. 2B). VEGF_188_ beads also attracted FBM neurons (Fig. 2B). Quantification confirmed that FBM neuron migration was significantly enhanced on the hindbrain side containing a VEGF_164_- or VEGF_188_-soaked bead relative to the control side of the same hindbrain (Fig. 2C). VEGF_188_ can therefore promote NRP1-dependent neuronal migration similar to VEGF_164_.

We next examined FBM neuron migration *in vivo* by *Isl1 in situ* hybridisation. As previously shown (Schwarz et al., 2004), *Vegfa^120/120^* hindbrains demonstrated abnormal streaming of FBM neurons on the ventricular side and dumbbell-shaped nuclei on the pial side (Fig. 2D). By contrast, *Vegfa^188/188^* mice, which express only VEGF_188_, showed normal FBM neuron migration (Fig. 2D). Moreover, replacing one *Vegfa^120^* allele in *Vegfa^120/120^* mutants with the *Vegfa^188^* allele was sufficient to prevent FBM neuron defects (Fig. 2D). Unlike VEGF_120_, VEGF_188_ is therefore sufficient to direct NRP1-dependent neuronal migration.

### VEGF_188_ is sufficient to guide NRP1-dependent axon crossing at the optic chiasm

**Fig. 3.**
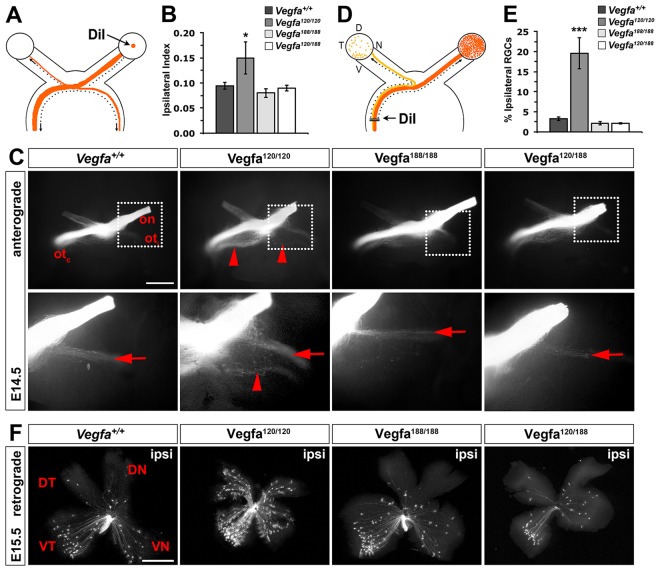
**VEGF_188_ is sufficient to guide commissural axons across the optic chiasm.** (A) Schematic illustration of the method used to anterogradely label RGC projections. DiI crystals were placed onto the retina in one eye to label axons extending through the optic chiasm into the ipsilateral and contralateral optic tracts. (B) Ipsilateral index in the indicated genotypes (mean±s.e.m.): control, 0.095±0.01, *n*=11; *Vegfa^120/120^*, 0.15±0.03, *n*=5; *Vegfa^188/188^*, 0.083±0.01, *n*=3; *Vegfa^120/188^*, 0.09±0.01, *n*=3; *t*-test, *P<0.05 compared with control. (C) Whole-mount views of RGC axons at the optic chiasm from embryos of the indicated genotypes, labelled anterogradely with DiI at E14.5; ventral view, anterior upwards; optic nerve (on), contralateral optic tract (ot_c_) and ipsilateral optic tract (ot_i_). Red arrows indicate the normal position of the ipsilateral projection; red arrowheads indicate the secondary tract and axon defasciculation in *Vegfa^120/120^* mutants. Scale bar: 500 µm. Higher magnifications of each boxed areas are shown beneath the respective panels. (D) Schematic illustration of the method used to retrogradely label RGC projections. DiI crystals were placed unilaterally into the optic tract in the dorsal thalamus. (E) Proportion of ipsilaterally projecting RGCs relative to total number of RGCs in both eyes of the indicated genotypes at E15.5 (mean±s.e.m.): control, 3.28±0.44%, *n*=8; *Vegfa^120/120^*, 19.64±3.89%, *n*=4; *Vegfa^188/188^,* 2.16±0.42%, *n*=4; *Vegfa^120/188^*, 2.12±0.14%, *n*=2; *t*-test, ***P<0.001 compared with control. (F) Flatmounted ipsilateral retinas from E15.5 embryos of the indicated genotypes after retrograde labelling from the optic tract in the right thalamus. DT, dorsotemporal; VN, ventronasal; DN, dorsonasal; VT, ventrotemporal. Scale bar: 500 µm.

We next investigated whether VEGF_188_ can evoke neuronal responses similar to VEGF_164_ in the developing visual system. To establish binocular vision, RGC axons project through the optic chiasm to both the ipsilateral and contralateral brain hemispheres (Erskine and Herrera, 2007). VEGF_164_, but not VEGF_120_, promotes RGC axon guidance in a NRP1-dependent fashion *in vitro*, and *Vegfa^120/120^* mice therefore develop an abnormal chiasm (Erskine et al., 2011). To examine whether VEGF_188_ can also promote RGC axon guidance, we performed DiI labelling in VEGF isoform mutants. Anterograde labelling of RGC axons from one eye at E14.5 demonstrated that VEGF_188_ was sufficient for NRP1-mediated chiasm patterning (Fig. 3A). Thus, *Vegfa^120/120^* mice had a significantly increased ipsilateral projection index as well as defasciculation of the ipsilateral and contralateral optic tracts (Erskine et al., 2011), but the ipsilateral index and shape of the optic chiasm appeared unaffected in *Vegfa^188/188^* mice (Fig. 3B,C). Moreover, replacing one *Vegfa^120^* with the *Vegfa^188^* allele was sufficient to prevent chiasm defects in *Vegfa^120/120^* mutants (Fig. 3B,C).

We next performed retrograde DiI labelling of RGC axons from the dorsal thalamus in VEGF isoform mice and compared the number of labelled RGCs in flatmounted ipsilateral and contralateral retina (Fig. 3D). Quantitation showed that the proportion of DiI-labelled ipsilateral RGCs was significantly increased in *Vegfa^120/120^* compared with control mice, but was normal in *Vegfa^188/188^* and *Vegfa^120/188^* mice (Fig. 3E). Flat-mount images also revealed the preferential origin of ipsilaterally projecting neurons from the ventrotemporal retina in wild types (Fig. 3F). Their distribution is affected in *Vegfa^120/120^* mice, which contain ipsilaterally projecting RGCs throughout the nasal retina (Erskine et al., 2011), but this defect was rescued by the introduction of a single *Vegfa^188^* allele (Fig. 3F). VEGF_188_ is therefore sufficient to promote NRP1-dependent aspects of optic chiasm development.

### VEGF_188_ is sufficient to ensure normal GnRH neuron survival

**Fig. 4.**
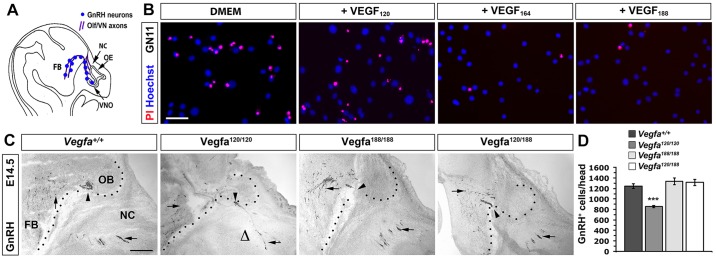
**VEGF_188_ is sufficient to promote GnRH neuron survival.** (A) GnRH neuron migration (blue dots). The neurons are born in the nasal placodes that give rise to the olfactory and vomeronasal epithelia (OE, VNO) and migrate along olfactory and vomeronasal axons (purple, Olf/VN) through the nasal compartment (NC) to reach the forebrain (FB). (B) Serum-starved GN11 cells were treated with DMEM or DMEM-containing serum, VEGF_120_, VEGF_164_ or VEGF_188_; cell death was visualised by propidium iodide staining (red); Hoechst staining (blue) identified the total number of cells. Scale bar: 25 µm. (C) Sagittal sections of E14.5 mouse heads of the indicated genotypes, immunolabelled for GnRH. Arrows indicate migrating neurons; arrowheads indicate blood vessels; open triangles indicate the absence of migrating neurons; dotted lines indicate the FB boundary. OB, olfactory bulb. Scale bar: 100 µm. (D) GnRH neuron number in E14.5 heads of the indicated genotypes (mean±s.e.m.): control, 1246±46, *n*=6; *Vegfa^120/120^*, 854±21, *n*=5; *Vegfa^188/188^*, 1335±63, *n*=3; *Vegfa^120/188^*, 1314±58, *n*=3; *t*-test; ***P<0.001 compared with control.

As a third model to study VEGF_188_ in neurodevelopment, we investigated GnRH neuron survival. GnRH neurons are born in the nasal placode and travel along nasal axons to reach the forebrain (Fig. 4A; Cariboni et al., 2007). We have previously shown that *Vegfa^120/120^* mice have significantly fewer migrating GnRH neurons and demonstrated that VEGF_164_ signals through NRP1 to promote the survival of GN11 cells, which recapitulate many features of migratory GnRH neurons (Cariboni et al., 2011). We therefore examined whether VEGF_188_ promotes GN11 survival, similar to VEGF_164_. Whereas 72 h of serum withdrawal caused the death of over half of the GN11 cells, the inclusion of serum, VEGF_164_ or VEGF_188_ for the last 12 h of culture significantly reduced cell death, and VEGF_188_ was as effective as VEGF_164_ in preventing cell death; by contrast, and as expected, VEGF_120_ did not promote survival (Fig. 4B; percentage of propidium iodide-positive cells, mean±s.e.m.: control, 44±3%; serum, 2±1%; VEGF_120_, 37±3; VEGF_164_, 11±2%; VEGF_188_, 11±2%). These observations suggest that VEGF_188_, similar to VEGF_164_, can promote GnRH neuron survival. The ineffectiveness of VEGF_120_ agreed with the previously observed NRP1-dependent neuroprotection of GN11 cells and the fact that *Vegfa^120/120^* mice have fewer GnRH neurons (Cariboni et al., 2011). Also in agreement with the *in vitro* findings, the GnRH neuron number was normal in *Vegfa^188/188^* mice that express VEGF_188_ but lack VEGF_164_ (Fig. 4C,D). Moreover, replacing one *Vegfa^120^* allele in *Vegfa^120/120^* mutants with the *Vegfa^188^* allele was sufficient to prevent their GnRH neuron survival defect (Fig. 4C,D). Together, these data show that VEGF_188_ is sufficient to promote NRP1-dependent neuronal survival.

### Conclusions

Our study has demonstrated that human VEGF_189_, but not VEGF_121_, binds NRP1 in a tissue context, that mouse VEGF_188_ is co-expressed with VEGF_164_ in a neuronal context, and that mouse VEGF_188_ expressed from the endogenous *Vegfa* locus can evoke NRP1-dependent neuronal responses *in vitro* and *in vivo*, similar to VEGF_164_ and unlike VEGF_121_. Future work on the role of VEGF signalling through NRP1, especially studies using *Vegfa^120/120^* or tissue-specific *Vegfa*-null alleles, should therefore consider the possibility that VEGF_188_, similar to VEGF_164_, can regulate the process under investigation. This consideration would be relevant for both neural and vascular studies, or indeed any context in which VEGF signalling through NRP1 is implicated. The finding that the relatively understudied VEGF_189_ is capable of evoking VEGF isoform-specific signalling events may have broad implications for the therapeutic use of VEGF. Thus, VEGF application has been considered in many studies for pro-angiogenic, pro-neurogenic and neuroprotective therapies, e.g. the treatment of amyotrophic lateral sclerosis (reviewed by Storkebaum et al., 2011). Most prior studies have used VEGF_165_ to ensure comprehensive receptor targeting; however, the retention of VEGF_165_ in tissues is inferior to that of VEGF_189_ due to the presence of only one instead of two heparin/matrix-binding domains. Our work demonstrating that VEGF_189_ is fully capable of engaging NRP1, in addition to its known ability to bind VEGFR1 and VEGFR2, therefore suggests that VEGF_189_ may be better suited than VEGF_165_ to induce localised tissue effects in therapeutic applications.

## MATERIALS AND METHODS

### Animals

Animal procedures were preformed in accordance with institutional and UK Home Office guidelines. The *Vegfa^120^* and *Vegfa^188^* alleles (Carmeliet et al., 1999; Stalmans et al., 2002), and *Nrp1^−/−^* and *Nrp2^−/−^* mice have been described previously (Giger et al., 2000; Kitsukawa et al., 1997).

### RT-PCR and sequencing

Total RNA was reverse transcribed using Superscript III (Life Technologies) and *Vegfa* isoforms amplified by PCR using MegaMix (Microzone) and the following oligonucleotide pairs: 120-F 5′-GTAACGATGAAGCCCTGGAG-3′ and 120-R 5′-CCTTGGCTTGTCACATTTTTC-3′; 164-F 5′-AGCCAGAAAATCACTGTGAGC-3′ and 164-R 5′-GCCTTGGCTTGTCACATCT-3′; 188-F 5′-AGTTCGAGGAAAGGGAAAGG-3′ and 188-R 5′-GCCTTGGCTTGTCACATCT-3′.

### AP-fusion protein binding assays

Open reading frames for the VEGF isoforms were amplified by PCR with the oligonucleotides 5′-AATAATGGATCCGCACCCATGGCAGAAGG-AG-3′ and 5′-TATATGCTCGAGCTCACCGCCTCGGCTTGTC-3′. The PCR products were cloned into pAG3-AP containing an upstream in-frame AP cassette. Binding assay were performed as described previously (Fantin et al., 2013b).

### Immunolabelling and *in situ* hybridisation

Primary antibodies used were: rabbit anti-mouse GnRH (Immunostar, 20075, 1:1000), goat anti-rat NRP1 (R&D Systems, AF566, 1:100), rabbit anti-mouse TUJ1 (Covance, MRB-435p, 1:250) and mouse anti-rat ISL1 (DSHB, 39.4D5, 1:100). Secondary antibodies used were: Alexa594-conjugated rabbit anti-goat Fab (Jackson ImmunoResearch, 305-587-003, 1:200), Alexa488-conjugated donkey anti-rabbit Fab (Jackson ImmunoResearch, 711-547-003, 1:200), Alexa488-conjugated goat anti-mouse (Life Technologies, A-110011, 1:200) and biotinylated goat anti-rabbit (Vector Laboratories, BA-1000, 1:200). To detect blood vessels, we used biotinylated IB4 (Sigma) followed by Alexa633-conjugated streptavidin (Life Technologies). For *in situ* hybridisation, we used a digoxigenin-labelled *Isl1* probe (Schwarz et al., 2004).

### Hindbrain explant culture

Hindbrain explants were cultured as previously described (Schwarz et al., 2004; Tillo et al., 2014). Affi-Gel heparin beads (Bio-Rad) were soaked overnight in 100 ng/ml of VEGF_164_ in PBS (Preprotech) or VEGF_188_ (Reliatech). FBM neuron migration was measured with ImageJ (NIH) as the distance travelled from r5 to the leading group of cells in r6 in each hindbrain and normalised to the control side of each hindbrain.

### DiI labelling

DiI labelling was performed with fixed tissues as described previously (Erskine et al., 2011). Briefly, a DiI crystal (Life Technologies) was placed over the optic disc of one eye for anterograde labelling. After 3 days at 37°C, dissected brains were imaged ventral side upwards. ImageJ was used to determine the pixel intensity in defined areas of the ipsilateral and contralateral optic tracts, and the ipsilateral index calculated as the ratio of fluorescent intensity in the ipsilateral relative to the ipsilateral plus contralateral tracts. For retrograde labelling, the cortex was removed unilaterally and DiI crystals placed in a row over the dorsal thalamus for 15 weeks at room temperature; we imaged flatmounted retinas as above and determined the percentage of labelled ipsilateral RGCs relative to the ipsilateral plus contralateral RGCs.

### GnRH neuron analysis and survival assays

Immunolabelled GnRH-positive cells were quantitated and GN11 survival assays performed as described previously (Cariboni et al., 2011). For survival assays, cells were serum starved for 72 h and treated for 12 h with media containing 10% FBS, 10 ng/ml VEGF_120_, VEGF_164_ or VEGF_188_.

## Supplementary Material

Supplementary Material
