# Supplementary material for: VEGF189 binds NRP1 and is sufficient for VEGF/NRP1-dependent neuronal patterning in the developing brain
Source: Development. 2015 Jan 15;142(2):314–9. doi: 10.1242/dev.115998 (PMC4302834; doi:10.1242/dev.115998)
Supplement: Supplementary Material [file supp_142_2_314__index.html]

Supplementary Material 

# VEGF189 binds NRP1 and is sufficient for VEGF/NRP1-dependent neuronal patterning in the developing brain

## DEV115998 Supplementary Material

**Files in this Data Supplement:**

- Supplementary Material
