# Supplementary material for: VEGF189 binds NRP1 and is sufficient for VEGF/NRP1-dependent neuronal patterning in the developing brain
Source: Development. 2015 Jan 15;142(2):314–9. doi: 10.1242/dev.115998 (PMC4302834; doi:10.1242/dev.115998)
Supplement: Supplementary Material [file supp_dev.115998_DEV115998supp.pdf]

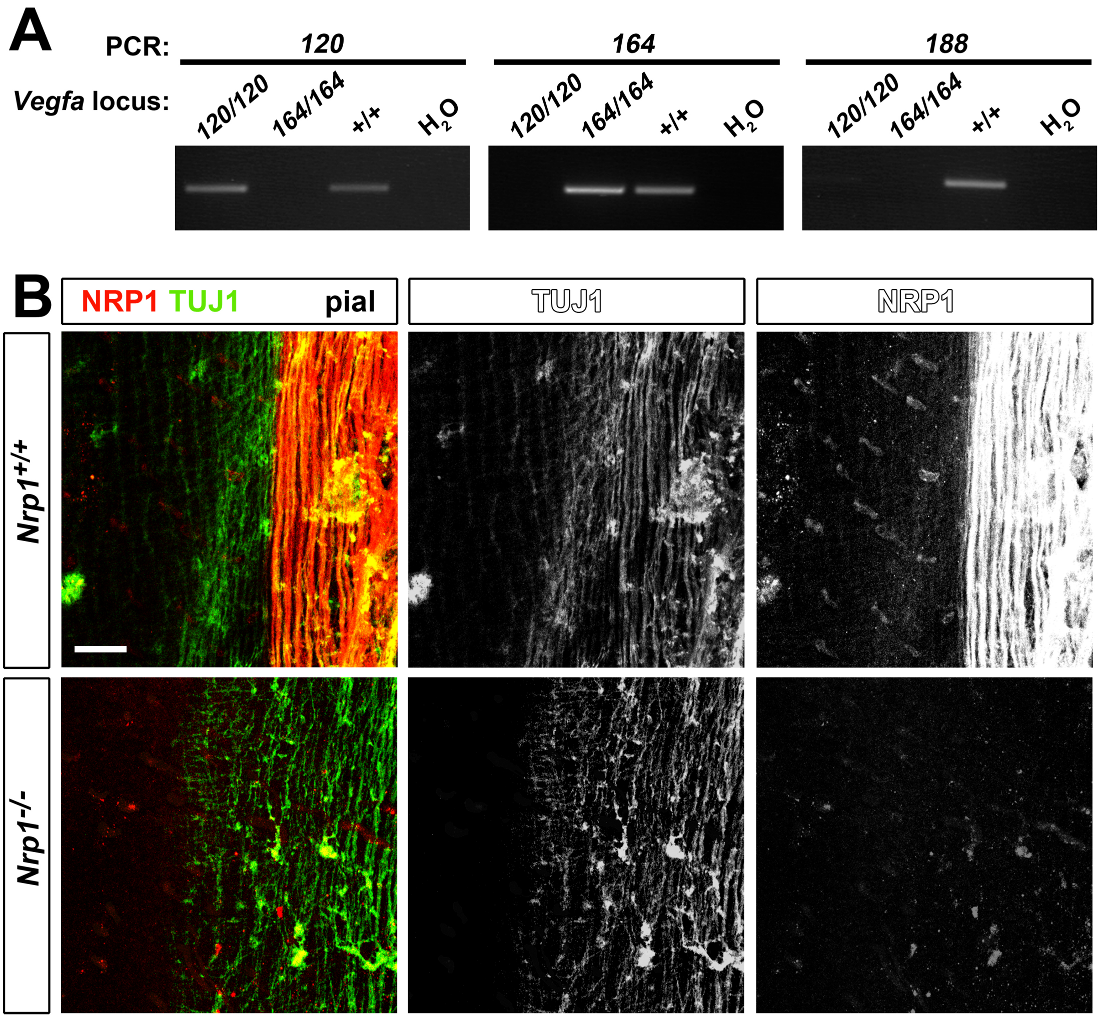

Fig. S1. ***Specificity of Vegfa isoform PCR reagents and perseverance of the dorsolateral fascicles in Nrp1-null mutants.***

(A) The specificity of oligonucleotide primers for *Vegfa* isoform expression analysis was validated by RT-PCR using cDNA derived from $Vegfa^{120/120}$, $Vegfa^{164/164}$ or wildtype E12.5 mouse embryo trunks, respectively. Note that a molecular weight standard confirmed the predicted sizes of each isoform as 179, 159 and 215 bp, respectively.

(**B**) Wholemount staining of E12.5 wildtype hindbrains for NRP1 and TUJ1; the single NRP1 and TUJ1 channels are shown in grey scale adjacent to each panel. Scale bar: 200 µm.
